# Immunity to melanin and to tyrosinase in melanoma patients, and in people with vitiligo

**DOI:** 10.1186/1472-6882-12-109

**Published:** 2012-07-26

**Authors:** Marija Đorđić, Ivana Z Matić, Ivana Filipović-Lješković, Radan Džodić, Miomir Šašić, Aleksandra Erić-Nikolić, Ana Vuletić, Branka Kolundžija, Ana Damjanović, Nađa Grozdanić, Srđan Nikolić, Janko Pralica, Danijela Dobrosavljević, Sanvila Rašković, Slađana Andrejević, Zorica Juranić

**Affiliations:** 1 Institute of Oncology and Radiology of Serbia, Pasterova 14, Belgrade, Serbia; 2 School of Medicine, University of Belgrade, Belgrade, Serbia; 3 Clinic of Dermatovenereology, Clinical Center of Serbia, Belgrade, Serbia; 4 Institute of Immunology and Allergology, Clinical Center of Serbia, Belgrade, Serbia

**Keywords:** Melanin, Tyrosinase, Melanoma, Vitiligo, Anti-tyrosinase antibodies, Anti-melanin antibodies, CD16+ CD56+, CD89+

## Abstract

**Background:**

The aim of this study was to determine the presence and the intensity of humoral immunity to melanoma-associated antigens: tyrosinase and melanin, in patients with melanoma, in persons with vitiligo and in control healthy people.

**Methods:**

The study involved 63 patients with melanoma and 19 persons with vitiligo. Control group consisted up to 41 healthy volunteers. Mushroom tyrosinase and synthetic melanin were used as the antigens.

**Results:**

ELISA test showed significantly (p < 0.0000004 and p < 0.04) lower levels of IgM anti-tyrosinase autoantibodies, in melanoma and vitiligo patients respectively, compared to controls.

Although there was no significant difference between the levels of IgA anti-melanin autoantibodies in melanoma or vitiligo patients in comparison with controls, the enhanced concentrations of anti-melanin IgA autoantibodies were preferentially found in melanoma patients with metastatic disease. Significantly high percentage in the Fc alphaRI (CD89) positive cells was determined in melanoma patients (p < 0.002 and p < 0.008) in comparison to that found in healthy people or in patients with vitiligo, in the already mentioned order, pointing that IgA dependent cellular cytotoxicity is not important for the immune action against melanoma, even more that it is included in some immune suppression.

Levels of IgG autoantibodies to mentioned antigens in melanoma patients although low were not significantly lower from controls. These findings analyzed together with the statistically significant low percentage of FcgammaRIII, (CD16) positive immunocompetent cells (p < 0.0007 and p < 0.003), which was found in patients with melanoma compared with healthy or vitiligo people respectively, and statistically significant low percentage of (CD16 + CD56+) natural killer (NK) cells (p < 0.005) found in melanoma patients in comparison to healthy controls pointed to the low probability for anti-melanoma IgG mediated, antibody mediated cellular cytotoxicity, (ADCC) and NK cytotoxicity. Moreover the ratio of the percentages of granulocytes and percentage of lymphocytes was statistically higher in patients with melanoma in relation to healthy people as well as to people with vitiligo (p < 0.0007 and p < 0.05 respectively).

**Conclusion:**

Autoantibodies to tyrosinase and to melanin which are found even in healthy people, point that consummation of edible mushrooms that carry the antigen tyrosinase and melanin, could influence the humoral anti-melanoma immune response.

Levels of different immunoglobulin classes of anti-melanin and anti-tyrosinase antibodies varied depending on the presence and the stage of studied diseases. Besides, the statistically enhanced ratio of the percentages of granulocytes and percentage of lymphocytes, together with statistically decreased percentage of NK cells is found in analyzed melanoma patients.

## Background

It is well known that melanoma is highly immune dependent malignant disease. The data on the frequency of spontaneous tumor regressions [1] and the frequency of tumor regressions after clinically-available immunomodulatory therapies [2-7], as well as that melanoma is related to immunosuppression [8] point that this disease is mainly under the control of host immunity. Melanoma is very serious disease and it is important, to emphasize, to not overlook, that very recently, some new immunotherapy approaches for suppression of metastatic melanoma showed great achievement. These are: autologous TILs administered in conjunction with interleukin-2 following a lymphodepleting preparative regimen [4,9], while the others are based on the enhancement of the immune system function by blockade of the cytotoxic T-lymphocyte associated antigen-4 (CTLA-4) by the monoclonal antibody ipilimumab which is at the present approved by the United States Food and Drug Administration (FDA) for use in patients with unresectable melanoma [6,7,10]. Other monoclonal antibodies targeting T-cell ligands, such as programmed death-1 (PD-1), also show promise. New biological agents designed to block oncogenic signal transduction such is vemurafenib inhibiting v-Raf murine sarcoma viral oncogene homologue B1 is active only in melanoma patients with tumor cells harboring BRAF mutations. It is considered for the therapy of melanoma alone as well as in combination with peptide vaccines or with ipilimumab, or with dacarbazine [11]. These very remarkable therapeutical approaches, as well as some new successful vaccination protocols offer new hope to patients with melanoma and now they are applied only in patients with metastatic disease [12]. On the other side, it is important to note that immunologically opposite disease, vitiligo, (a dermatological disorder characterized by the loss of melanin, which results in depigmented areas of the skin), appears in many cases of melanoma regression [13,14].

It was reported that lysates of melanoma cells [15-17], peptides derived from these antigens, irradiated autologous or allogenous melanoma cells [18], are used for immunization against melanoma.

The question arises are some other sources of melanoma-associated antigens e.g. melanin or the key molecule in its biosynthesis - tyrosinase (from edible mushrooms), immunogenic in melanoma patients, in people with vitiligo, and in healthy people? If the answer is “Yes”, it needs to be checked whether these antigens could be used in the form of everyday meals for the induction of adaptive immunity in group of melanoma patients without metastatic disease, who are without therapy after the surgical removal of their tumors.

In our previous articles we proved that many of food antigens (like gliadin from wheat, cow’s milk proteins, phytohemagglutinin from red beans), induced some kind of immune-mediated molecules - synthesis of various class of immunoglobulins, IgG, IgA, IgM [19-21]. Similarly to this it was easy to propose that the edible mushrooms as the rich source of tyrosinase and of melanin, after the consummation, could induce immunity to mentioned molecular structures.

The aim of this study was to determine the presence and the intensity of humoral immunity to melanoma-associated antigens: melanin and tyrosinase in patients with melanoma, in people with vitiligo and in control healthy people. In that sense, the levels of serum IgM, IgG, and IgA immunity to tyrosinase and to melanin, as well as the percentage of cells which could be included in immune antibody- dependent cellular cytotoxicity (ADCC), -of FcgammaRIII positive immunocompetent cells CD16+, and percentage of NK (CD16+ CD56+) positive lymphocytes as well as Fc alphaRI positive (CD89+) cells , in patients with melanoma, and in people with vitiligo, were determined and analyzed.

## Results

**Figure 1:**
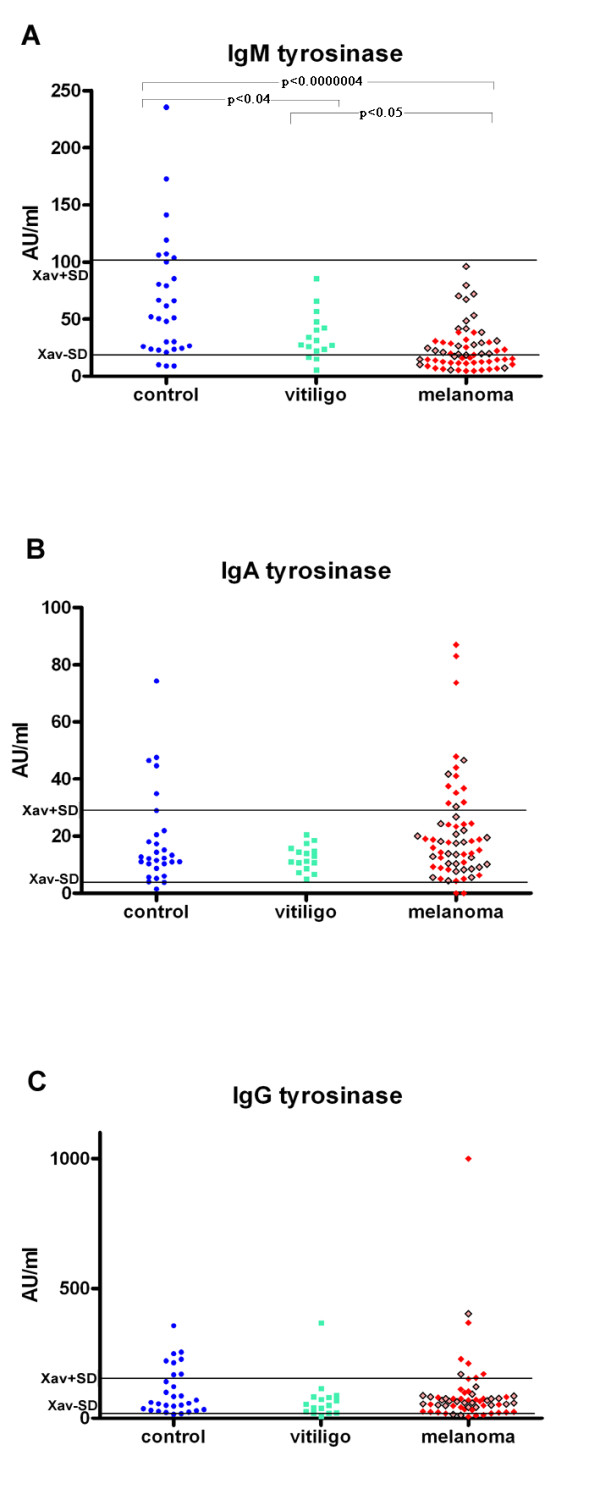
**(A) Serum IgM immunoreactivity to mushroom tyrosinase in healthy controls and in patients with melanoma or vitiligo, (B) Serum IgA immunoreactivity to mushroom tyrosinase in healthy controls and in patients with melanoma or vitiligo, (C) Serum IgG immunoreactivity to mushroom tyrosinase in healthy controls and in patients with melanoma or vitiligo.** Red squares represent melanoma patients with metastatic disease, while brighter red squares with borders represent melanoma patients without metastases.

The significantly lower levels of IgM anti-tyrosinase autoantibodies are found in melanoma patients and in people with vitiligo, in comparison to that found in controls p < 0.0000004 and p < 0.04 respectively, as seen on Figure 1. (Moreover 24 out from 28 melanoma patients which had decreased anti-tyrosinase IgM autoantibodies had metastatic disease as seen on Table 1 and Figure 1.). It was also found that the levels of mentioned antibodies in melanoma patients were statistically significantly lower in comparison to that found in vitiligo patients (p < 0.05), Figure 1. There was no statistically significant difference between levels of IgA or IgG anti-tyrosinase autoantibodies in patients with melanoma and in patients with vitiligo compared to controls.

**Table 1:** Frequencies of people with disturbed levels of anti-melanin and anti-tyrosinase autoantibodies

	**Healthy control people**	**People with vitiligo**	**Melanoma patients**	**Melanoma patients (without metastases)**	**Melanoma patients (with metastases)**
**Increased anti-melanin IgM**	7 (32)	2 (16)	3 (62)	3 (27)	0 (35)
**Decreased anti-melanin IgM**	4 (32)	2 (16)	11 (62)	4 (27)	7 (35)
**Increased anti-melanin IgA**	5 (32)	0 (16)	22 (62)	7 (27)	15 (35)
**Decreased anti-melanin IgA**	2 (32)	1 (16)	13 (62)	5 (27)	8 (35)
**Increased anti-melanin IgG**	7 (32)	3 (16)	13 (62)	9 (27)	4 (35)
**Decreased anti-melanin IgG**	5 (32)	5 (16)	6 (62)	2 (27)	4 (35)
**Increased anti-tyrosinase IgM**	7 (30)	0 (16)	0 (62)	0 (26)	0 (36)
**Decreased anti-tyrosinase IgM**	3 (30)	3 (16)	28 (62)	4 (26)	24 (36)
**Increased anti-tyrosinase IgA**	5 (29)	0 (16)	14 (62)	3 (26)	11 (36)
**Decreased anti-tyrosinase IgA**	2 (29)	0 (16)	2 (62)	0 (26)	2 (36)
**Increased anti-tyrosinase IgG**	8 (29)	1 (16)	7 (62)	2 (26)	5 (36)
**Decreased anti-tyrosinase IgG**	1 (29)	1 (16)	5 (62)	2 (26)	3 (36)

**Figure 2:**
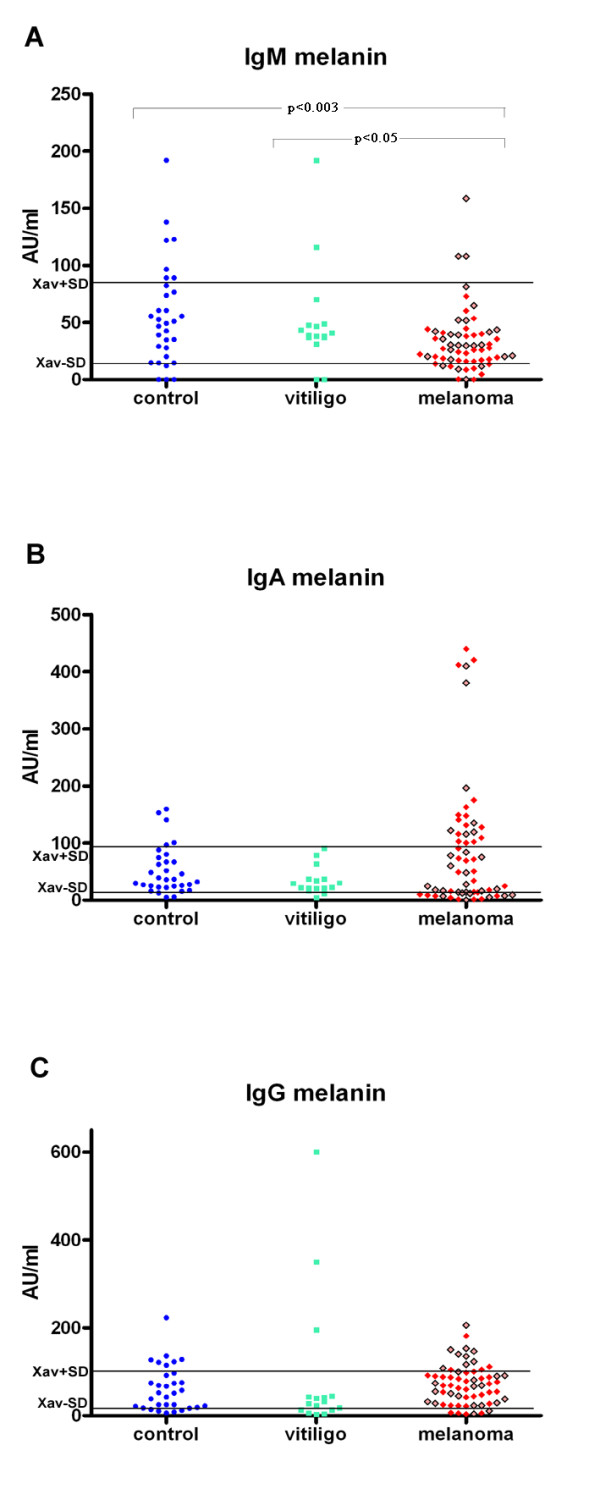
**(A) Serum IgM immunoreactivity to melanin in healthy controls and in patients with melanoma or vitiligo, (B) Serum IgA immunoreactivity to melanin in healthy controls and in patients with melanoma or vitiligo, (C) Serum IgG immunoreactivity to melanin in healthy controls and in patients with melanoma or vitiligo.** Red squares represent melanoma patients with metastatic disease, while brighter red squares with borders represent melanoma patients without metastases.

Statistically significant low level of anti-melanin IgM autoantibodies was found in melanoma patients in comparison to healthy control group (p < 0.003) and to people with vitiligo (p < 0.05) as seen on Figure 2. Although there was no statistically significant difference in the levels of anti-melanin IgA autoantibodies in melanoma patients compared with controls, 15 out from 22 melanoma patients with enhanced levels of IgA anti-melanin autoantibodies had metastatic disease (Table 1 and Figure 2).

Most of melanoma patients had low levels of IgG anti-melanin autoantibodies. The low levels of anti-melanin IgG autoantibodies were also found in some patients with vitiligo in comparison to healthy controls (Figure 2).

**Figure 3:**
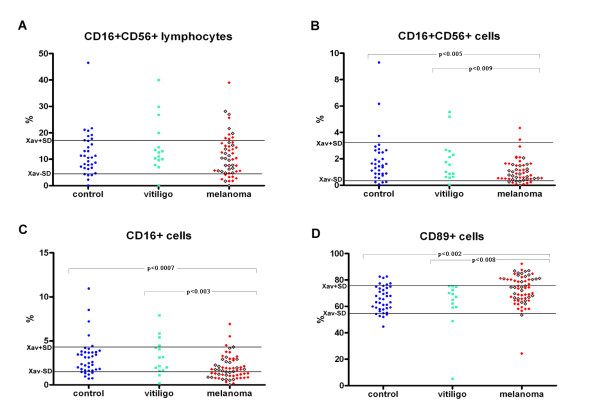
**(A) Percentage of CD16 + CD56+ lymphocytes (NK cells) in healthy controls and in patients with melanoma or vitiligo, (B) Percentage of CD16 + CD56+ overall white blood cells (NK cells) in healthy controls and in patients with melanoma or vitiligo, (C) Percentage of CD16+ overall white blood cells in healthy controls and in patients with melanoma or vitiligo, (D) Percentage of CD89+ overall white blood cells in healthy controls and in patients with melanoma or vitiligo.** Red squares represent melanoma patients with metastatic disease, while brighter red squares with borders represent melanoma patients without metastases.

As presented on Figure 3 there was a statistically significant decrease in the percentage of FcgammaRIII, (CD16) positive immunocompetent cells (p < 0.0007 and p < 0.003), in patients with melanoma compared with healthy or vitiligo people respectively. The significantly low percentage of NK (CD16 + CD56+) cells (p < 0.005) was found in melanoma patients in comparison to healthy controls.

**Figure 4:**
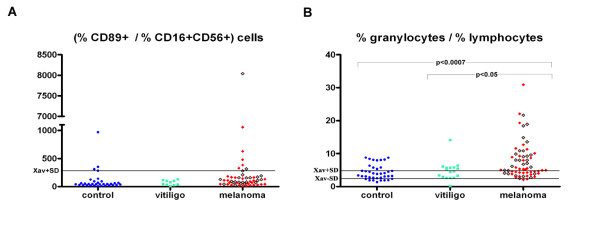
**(A) Ratio of the percentage of CD89+ overall white blood cells and the percentage of CD16 + CD56+ overall white blood cells (NK cells) in healthy controls and in patients with melanoma or vitiligo, (B) Ratio of the percentage of granulocytes and the percentage of lymphocytes in healthy controls and in patients with melanoma or vitiligo.** Red squares represent melanoma patients with metastatic disease, while brighter red squares with borders represent melanoma patients without metastases.

From Figure 4 it could be seen that the ratio of the percentages of granulocytes and percentages of lymphocytes is statistically higher in patients with melanoma in relation to healthy people as well as to people with vitiligo ( p < 0.0007 and p < 0.05 respectively).

**Figure 5:**
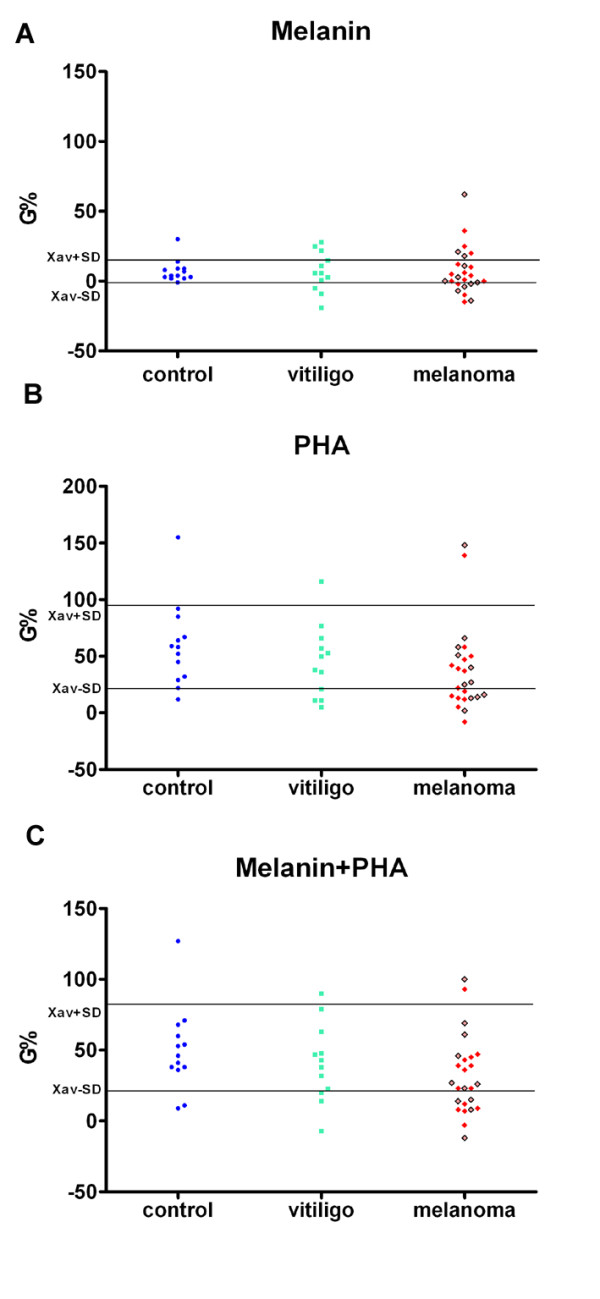
***In vitro *stimulation (G%) of PBMC of healthy controls, as well as of patients with malanoma or vitiligo, on proliferation by synthetic melanin (5 μg/ml) (A), or by phytohemagglutinin (5 μg/ml) from red beans (B), or by the mixture of melanin and phytohemagglutinin (C), in nutrient medium RPMI 1640 with 10 % autologous plasma.** Red squares represent melanoma patients with metastatic disease, while brighter red squares with borders represent melanoma patients without metastases.

Mild, but not significant decrease in PBMC survival (induced by tumor antigen melanin) was found in few melanoma patients. The higher but not statistically significant stimulation of PBMC by tumor antigen melanin and PHA was found in healthy people compared to patients with melanoma (Figure 5).

## Discussion

The immunogenic role of tyrosinase in melanoma has been already proved, and results presented in this work are in accordance with previously published papers on the presence of anti-tyrosinase antibodies in the serum of control people as well as in patients with melanoma or vitiligo [22-24]. The direct importance of immunity to mushroom tyrosinase for the melanoma disease is obtained from the study in which it is reported that mice immunized with mushroom tyrosinase generated a high titer of anti-tyrosinase antibodies which after the inoculation of melanoma cells developed a lower number of lung metastases compared with an unvaccinated control group [23].

Melanin is an antigen whose role in immune control of melanoma is proved *in vivo*. It is important to note that although melanin is an intracellular pigment, anti-melanin IgM antibodies labeled with (188) Re were reported to be successful in directed radionuclide to melanoma tumor, in radioimmunotherapy of experimental metastatic melanoma [25].

New in this work are the data that the different levels of immunoglobulin isotypes (IgM, IgA or IgG) are found in melanoma or vitiligo patients compared with controls.

The statistically significant low levels of IgM anti-tyrosinase and IgM anti-melanin autoantibodies in melanoma patients and slight elevation in IgM anti-melanin autoantibodies in patients with vitiligo compared to healthy controls, point to the importance of IgM autoantibodies for both: the control of malignant disease, as well as for the destruction of melanocytes in vitiligo.

Enhanced levels of anti-melanin IgA autoantibodies which are preferentially found even in the presence of normal levels of FcAlphaRI (CD89) positive immunocompetent cells, in majority of melanoma patients with metastatic disease point to their disability in immunological suppression of malignant process (through ADCC) and even indicate their blocking –immunosuppressive action. This result appears to be an explanation of the findings reported earlier that the intensity of anti-melanoma cell-mediated cytotoxicity in melanoma patients, in the presence of autologous serum, was significantly lower in comparison to that found in control subjects and vitiligo patients [26]. This was attributed to the presence of some factors from melanoma patient's sera which contribute to impairment of the cytotoxicity of autologous PBMC, while other factors from the serum of vitiligo patients and control subjects enhanced their PBMC anti-melanoma cytotoxicity. In the light of results of this work, these factors present in melanoma patient's sera could be the blocking IgA anti-melanin antibodies, which are found in majority of melanoma patients, and the absence (lower levels) of these IgA antibodies from the vitiligo patients and control subject’s sera.

The lower percentage of NK cells, and of the percentage of CD16 positive immunocompetent cells in melanoma patients found in this work, point to the already known deficiency of these cell subpopulations and to the suppressed role of NK and of IgG mediated ADCC in the antitumor immunity in melanoma patients [27,28].

But it need to be emphasized that the ratio of the percentages of granulocytes and percentages of lymphocytes is statistically higher in patients with melanoma in relation to healthy people as well as to people with vitiligo.

Healthy person with the highest anti-melanin IgM level reported that she consumed approximately 100 g of edible mushrooms twice weekly, while the other one with the highest anti-melanin IgG level consumed approximately 100 g of edible mushrooms twice monthly and took additionally, every day 4 mg of the antioxidant astaxanthin (Oriflame).

In accordance to the presented data, results from this work set up the question whether the additional simple approach – a diet consisting of the consummation of 50 g cooked edible mushrooms twice weekly in the meal, along with prescribed oncological therapy, might induce the appropriate effective immune response: anti-melanin or anti-tyrosinase IgM or IgG in melanoma patients patients (with low percentage of granulocytes) in order to prevent metastatic disease? Undesired type of enhance blocking- IgA immunity was shown to be possible to be downregulated by the consumption of cocoa [29,30], while antioxidant astaxanthin enhanced IgM and IgG biosynthesis, as it was shown *in vivo*[31]; the therapy with Rituximab might also be taken into consideration [32].

Till now the role of nutrition in the control of melanoma was already reported. Recent results from a randomized phase II trial in melanoma patients indicate a significant benefit for patients treated with dacarbazine in combination with fermented wheat germ extract in terms of progression free survival and overall survival [33].

The use of diclofenac and nimesulide could be the option for in the inflammation suppression as it was shown that these agents do not have adverse effects; they do not stimulate *in vitro* B16F1 melanoma cell proliferation [34]. From the other side, as the neutrophil activation is implicated in the pathogenesis of inflammatory processes, the use of the known antioxidant and inhibitor of neutrophil respiratory burst N- acetylcysteine may be taken into consideration as the better option for the inflammation suppression [35].

## Conclusions

Autoantibodies to tyrosinase and to melanin which are found even in healthy people, point that consummation of edible mushrooms that carry the antigen tyrosinase and melanin, could influence the humoral anti-melanoma immune response.

Levels of different immunoglobulin classes varied depending on the presence and the stage of studied diseases. Besides, the statistically enhanced ratio of the percentages of granulocytes and percentage of lymphocytes together with statistically decreased percentage of natural killer (NK) cells found in analyzed melanoma patients points to the need of the therapeutic approach which could combine not only antigen stimulation , but also therapy whose action should be to decrease the inflammation- to decrease percentage of granulocytes.

## Methods

### Patients

The study involved 63 patients with melanoma not treated by any type of oncological therapy, even before surgical resection of the tumor and 19 patients with vitiligo. Obtained tissue samples of melanoma patients were cytologically and pathohistologically examined. It should be noted that 36 melanoma patients were with metastatic disease. Control group consisted of 32 and 30 healthy volunteers for testing immunoreactivity to melanin or tyrosinase respectively. The protocol of the study was approved by the Ethics Committee of the Institute of Oncology and Radiology of Serbia and by the Ethics Committee of Clinical Center of Serbia. Written informed consent was obtained from each patient.

### ELISA tests

The levels of serum anti-melanin, or anti-tyrosinase IgA, IgG and IgM autoantibodies were determined by ELISA [21]. (Two forms of tyrosinase exist: intracellular membrane bound form -consisting of inner, transmembrane and cytoplasmic domain-, and soluble form. It is proposed that membrane soluble forms could serve as an antigen. Tyrosinase is detected in the serum as well). In addition, melanocytes possess phagocytic activity and express MHC II molecules, therefore can present antigens derived from tyrosinase and melanin [24]. Assuming that some immunogenic epitopes are the same in the molecules of synthetic and in biosynthesized human melanin, and with the knowledge that mushroom and human tyrosinase share some same immunogenic determinants [22,23], synthetic melanin (SIGMA) and edible mushroom tyrosinase (SIGMA) were used as the antigens. (Mushroom tyrosinase, purchased from Sigma Aldrich, has been reported to have 16.32 % sequence identity and 41.08 % sequence similarity with human tyrosinase [24]). Concentrations of serum IgM, or IgA, or IgG, anti-melanin and anti-tyrosinase antibodies were expressed in AU/ml; human sera with the highest anti-melanin and anti-tyrosinase immunity were used for calibration. In the order to obtain clinically more useful data all values higher than Xav’+/−2.5SD, obtained analyzing the levels of anti-melanin and anti-tyrosinase immunity in healthy people were discarded for getting new Xav. Cut-off values for each anti-melanin or anti-tyrosinase immunoglobulins were (Xav ± SD) AU/ml.

### Flow cytometry analysis

In order to investigate is there any possibility for the ADCC and natural killer cytotoxic action, the flow cytometry was performed for analysis of CD89, and CD16 and CD16CD56 expression on granulocytes or on lymphocytes respectively. Monoclonal antibody specific for CD56 was FITC-stained, while monoclonal antibodies specific for CD16 and CD89 were PE-stained (Becton Dickinson Immunocytometry Systems, CA, USA). Cut-off values (Xav ± SD) were obtained for 41 healthy controls.

Expression of mentioned antigens on white blood cells was determined using a FACSCalibur flow cytometer (BD Biosciences Franklin Lakes, NJ, USA). Acquired data were analyzed using CELLQuest Software (BD Biosciences).

### Determination of PBMC stimulation

*In vitro* stimulation (G%) of peripheral blood mononuclear cells (PBMC) of healthy controls, as well as of patients with melanoma or vitiligo, on proliferation by synthetic melanin (5 μg/ml), or by non-specific lymphocyte stimulator phytohemagglutinin (5 μg/ml) from red beans, or by the mixture of melanin and phytohemagglutinin, in nutrient medium RPMI 1640 with 10 % autologous plasma was done using MTT test. This method based on the use of 3-(4,5-dimethylthiazol-2-yl)-2,5-diphenyltetrazolium bromide to assess the fraction of living cells by their mitochondrial dehydrogenase activity [36]. The principle of the tetrazolium-based tests is the conversion of the yellow tetrazolium salt by metabolically active live cells into a colored formazan dye.

### PBMC preparation

Briefly, PBMC were isolated by centrifugation in concentration gradient from the heparinized blood by separator (Lymphoprep^TM^, Nycomed, Oslo, Norway). PBMC are three times washed in Haemacel (aqueous solution of 145 mM Na^+^, 5.1 mM K^+^, 6.2 mM Ca^2+^, 145 mM Cl^−^, 35 g 1^1^ of gelatinous polymers, pH 7.4). Haemacel was then removed and the cells were resuspended in the nutrient medium (RPMI 1640, pH 7.2, supplemented with 10 % autologous plasma, 3 mM L-glutamine, 100 μg ml^-1^ streptomycin, 100 IU ml^-1^ penicillin and 25 mM Hepes).

These PBMC (150,000/well) were seeded and were incubated in 150 μl nutrient medium, in 96 microwell plates in the presence of melanin (5 μg/ml), PHA (5 μg/ml), or the mixture of melanin and PHA. Control PBMC were seeded in nutrient medium only. The incubation was ended 72 h later. Stimulation of PBMC to proliferation (G%) was determined as the increase in the number of cells in the presence of antigen(s) in comparison to the controls, PBMC incubated in nutrient medium only. As the numbers of cells is proportional to the absorbance of MTT treated cells G (%) was calculated as:

(1) $$\text{G}\left(\%\right)=\left(\text{A}-\text{Ao}\right)\times \frac{100}{\text{Ao}}$$

where A is the absorbance of PBMC incubated in medium with antigens, and Ao is the absorbance of control cells incubated in nutrient medium only.

### Statistical analysis

Two-tailed Student’s *T* test was used for statistical analysis of experimental data.

## Competing interests

The authors declare that they have no competing interests.

## Authors’ contributions

MĐ has done ELISA tests and flow cytometry, and performed data analyses. IM performed flow cytometry analyses, interpreted obtained data and technically prepared manuscript. AV performed flow cytometry. BK, AD, NG and IFLJ have done ELISA tests. RDž, MŠ, SN and JP enrolled patients with melanoma in the study and interpreted obtained data. SA, SR, DD enrolled people with vitiligo in the study and interpreted data. AEN helped with analysis of melanoma patients data. ZJ designed the study, interpreted data and wrote the first and last version of the manuscript. All authors read and approved the final version of the manuscript.

## Pre-publication history

The pre-publication history for this paper can be accessed here:

http://www.biomedcentral.com/1472-6882/12/109/prepub
